# Use of the Lüscher Color Test in Pediatric Dentistry: A Prospective Study in Behaviorally Challenging Pediatric Dental Patients Undergoing Conscious Sedation

**DOI:** 10.3390/children13030370

**Published:** 2026-03-05

**Authors:** Chiara Alessandra Dini, Maria Assunta Mauri, Lucia Giannini, Gregorio Menozzi, Giovanni Battista Grossi, Cinzia Maspero, Roberto Biagi

**Affiliations:** 1 Fondazione IRCCS Cà Granda Ospedale Maggiore Policlinico, 20122 Milan, Italy; chiara.dini@policlinico.mi.it (C.A.D.); gregorio.menozzi@unimi.it (G.M.); giovanni.grossi@unimi.it (G.B.G.); cinzia.maspero@unimi.it (C.M.); roberto.biagi@unimi.it (R.B.); 2 Department of Biomedical, Surgical and Dental Sciences, School of Dentistry, University of Milan, 20122 Milan, Italy; maria.mauri@unimi.it

**Keywords:** pediatric dentistry, dental anxiety, heart rate, psychological tests, color perception, nitrous oxide, procedural sedation, prospective studies

## Abstract

**Background:** Dental anxiety is common in pediatric dentistry and may hinder care, particularly in behaviorally challenging children. Most anxiety measures rely on verbal report, which can be unreliable in young patients. This study explored whether the Lüscher Color Test, a non-verbal psychological instrument, shows associations with established anxiety proxies in a pediatric dental sedation setting. **Methods:** In this single-center prospective observational study, 100 children aged 4–12 years referred for dental treatment in a conscious sedation unit were recruited; 80 completed the protocol (exclusion rate 20%). N_2_O/O_2_ inhalation sedation was not randomized and was selected by the clinician based on clinical judgement. Anxiety was assessed pre- and post-operative using the Lüscher Color Test, heart rate (HR) monitoring, and the Visual Facial Anxiety Scale (VFAS). The primary outcome was the pre–post change in the Lüscher anxiety index calculated as the pre-operative score minus the post-operative score (Δ = pre − post). Associations between changes in anxiety measures and demographic/clinical variables were examined. **Results:** Anxiety scores decreased after treatment for both the Lüscher Color Test and VFAS (both *p* < 0.001). Change in Lüscher scores was positively associated with HR reduction (Spearman r = 0.68; *p* < 0.01), whereas VFAS change showed a weaker association (r = 0.28; *p* < 0.05). In regression analyses, treatment-related variables were explored; however, given the observational design and subgroup imbalance, these findings should be interpreted cautiously. **Conclusions:** Although pre–post scores suggested a reduction in anxiety, the Lüscher Color Test should be considered an exploratory, complementary non-verbal measure rather than a validated diagnostic instrument. In the multivariable logistic regression, nitrous oxide sedation showed only a non-significant trend toward greater anxiety reduction (*p* = 0.07). Further studies with appropriate validation frameworks and stronger designs are needed before clinical implementation can be recommended.

## 1. Introduction

The World Health Organization recognizes dental phobia as a genuine clinical condition and, as such, should not be underestimated. In the International Classification of Diseases (ICD-10), it is classified as a specific phobia and encompasses the concepts of fear and anxiety. Dental anxiety is defined as a feeling of mental anguish, agitation, and restlessness experienced by the patient during the dental session and is an issue that is frequently encountered in dental practice [1]. The WHO estimates that 15–20% of the population with dental phobia are continually putting off treatment, relying on pharmacological therapies (antibiotics, painkillers), which only delay the resolution of the problem [2]. Many patients who attend the dentist suffer from a certain level of anxiety, and it is the dentist’s responsibility to manage this anxiety in a way that is appropriate and adequate for each patient, especially in pediatric patients [3]. In children, the presence of these emotions, often amplified by misconceptions and fears or by previous negative experiences, leads to the development of uncooperative attitudes, making treatment more difficult for the clinician. Numerous studies indicate that a child’s negative experiences of dental examinations and treatments during childhood will negatively influence his or her future experiences, becoming a real impediment to achieving good oral health.

For this reason, early diagnosis in pediatric patients is of fundamental importance to prevent, from the earliest approach, the development of negative emotions, thus laying the foundations for the promotion of good oral health [2,3]. In dental practice, anxiety can be assessed using different approaches, including patient interviews, behavioral observation, and a range of psychological instruments that measure either general anxiety or anxiety specifically related to dental care. The psychological and pedagogical sciences in pediatric dentistry use knowledge and techniques to assess, control, and modify uncooperative behavior with diagnostic elements [4,5]. Among the most widely used are the Facial Image Scale (FIS), the Visual Facial Anxiety Scale (VFAS), the Venham Test, the Modified Anxiety Scale (MCDAS), and the Frankl Behavior Rating Scale (FBRS). However, most of these methods for evaluating anxiety do not allow doctors to have completely objective results. Therefore, a suitable test for pediatric patients was sought—one that would be easy to apply, simple to administer, and capable of assessing the patient’s state of anxiety as objectively as possible.

In this context, rapid non-verbal assessment tools may offer potential advantages in pediatric settings.

Projective techniques such as the Lüscher Color Test have been historically used to explore emotional states through color preferences. Developed by Max Lüscher in 1947, the test requires individuals to rank colors according to preference, with the underlying assumption that color choices may reflect current emotional and psychophysiological conditions [6]. The test is brief (approximately 5–10 min) and does not require advanced verbal or cognitive skills, features that may be advantageous in young children [6,7]. However, the scientific status of the Lüscher Color Test remains debated: its psychometric reliability and construct validity are not consistently supported in the modern psychological literature, and it is not recommended as a stand-alone diagnostic tool. Furthermore, to date, its specific application in pediatric dental anxiety has been only marginally investigated.

According to Lüscher’s theoretical framework, while basic color perception is grounded in physiological sensory processing, individual color preferences are hypothesized to reflect underlying emotional states. However, empirical evidence suggests that color preferences and their interpretations may vary across populations and cultural contexts. Therefore, the universality proposed by the original model should be regarded as a theoretical assumption rather than an established fact, particularly within pediatric dental settings [7,8].

Given these considerations, the Lüscher Color Test should currently be viewed as a potentially informative but exploratory non-verbal measure rather than a validated diagnostic tool for dental anxiety. In pediatric dentistry, however, the availability of rapid, non-verbal screening instruments could be clinically valuable, especially for young or poorly cooperative children.

The aim of this study was to evaluate the applicability of the Lüscher Color Test in a pediatric dental conscious sedation clinic and to assess its associations with VFAS and heart rate measures before and after treatment. This work is intended as hypothesis-generating and does not constitute a formal validation of the test.

Primary objective: to evaluate the clinical applicability of the Lüscher Color Test for detecting pre- and post-operative anxiety in uncooperative pediatric dental patients.

Secondary objectives:

1. To assess the agreement between anxiety levels measured using the Lüscher Color Test and those obtained with the Visual Facial Anxiety Scale (VFAS) and heart rate monitoring.
2. To analyze changes in pre- and post-operative anxiety in relation to the following variables:
  - Age;
  - Sex;
  - Type of dental treatment (invasive vs. non-invasive);
  - Dental clinician;
  - Use of nitrous oxide inhalation sedation.

Invasive treatments: any procedure involving local anesthesia and/or operative tissue disruption. Non-invasive treatments: procedures without local anesthesia and without tissue disruption, limited to non-operative procedures and pharmacological management.

## 2. Materials and Methods

### 2.1. Sample Size

Sample size was determined a priori based on feasibility and expected recruitment over the study period (October–December 2019) in the Unit of Conscious Sedation. A target of 100 children was set to allow meaningful subgroup analyses while accounting for potential dropouts or incomplete data collection. No formal a priori power calculation was performed. Based on the final analyzable sample (n = 80), the study had approximately 80% power (two-sided α = 0.05) to detect at least a moderate correlation (≈r ≥ 0.31) between changes in Lüscher scores and physiological measures (heart rate reduction), which was one of the primary analytic aims.

### 2.2. Study Design

This study was conducted as a single-center prospective observational study on pediatric dental patients referred to the Pediatric Dentistry Unit of Fondazione IRCCS Cà Granda—Ospedale Maggiore Policlinico, Milan, Italy.

### 2.3. Participants

100 children aged between 4 and 12 years (with a mean age of 6.4 ± 1.6) were consecutively recruited among all children meeting the eligibility criteria and scheduled for dental treatment during the study period.

Children were classified as “uncooperative” based on the prior clinical judgment of the referring dentists. No standardized behavioral assessment scale (e.g., the Frankl Behavior Rating Scale) was systematically used at the time of referral.

The experimental setting was the Unit of Conscious Sedation. The evaluations were performed between October and December 2019.

Inclusion criteria:

The following inclusion criteria were applied:

- ASA I and II patients.
- Age between 4 and 12 years.
- Absence of contraindications to the administration of nitrous oxide.
- Patients whose parents provided written informed consent for participation.

Exclusion criteria

Patients were excluded from the study if they met any of the following criteria:

- Age outside the predefined range (younger than 4 years or older than 12 years);
- Presence of systemic conditions classified as ASA III or higher;
- Contraindications to N_2_O/O_2_ inhalation sedation;
- Inability to complete both the pre-operative and post-operative anxiety assessments;
- Absence or incomplete recording of heart rate data during the dental procedure;
- Need for urgent dental treatment that prevented adherence to the study protocol;
- Refusal to participate or lack of written informed consent from both parents or legal guardians.
- Out of a total of 100 patients in the present study sample, 20 were excluded because they did not meet the following inclusion criteria:
  - 7 were not within the age range studied.
  - 4 had not completed the second phase of the test.
  - 6 did not have heart rate recorded.
  - 3 had urgent admission for dentoalveolar trauma.

### 2.4. Treatments

The experimental protocol was carried out by the dentists of the Unit of Conscious Sedation, with the participation of a medical doctor specialized in drawing psychology and pedagogy.

#### 2.4.1. Operative Phase

Pre-operative anxiety assessment

Upon entering the clinic, the child was administered the Lüscher test according to the following protocol:

(a) A clinician performed the administration by arranging the 8 colored cards in a semicircle on a table.

The color cards were presented simultaneously and arranged randomly on a flat surface in front of the child. Children were instructed to select the colors as quickly and instinctively as possible to capture their immediate emotional response. No strict time limit was imposed, but prompt responses were encouraged.

**Figure A1:**
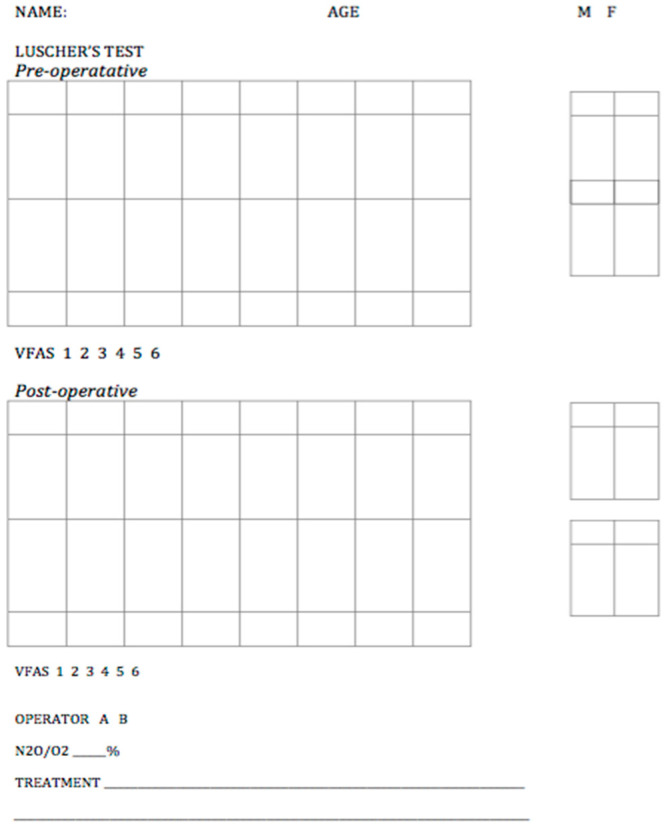
Data collection sheet created by the clinician for the tests administered: Lüscher and VFAS.

(b) The child was asked to select his or her preferred card from the eight available colors and place it upside down with the colored side facing the clinician. The child then continued selecting the remaining cards in order of preference.
(c) Reading from left to right the numbers on the back of the cards, the clinician recorded them in order on a card [Appendix A, Figure A1].
(d) After having shuffled them, the clinician arranged the cards again in front of the child.
(e) The child was asked to repeat the choice without trying to remember or reproduce what he had done previously. The second choice is usually made more spontaneously and is therefore considered more valid than the first, especially in doubtful cases.
(f) The second sequence was also recorded.
(g) Upon the Lüscher test administration, the VFAS was then shown to the children, who were then asked to choose the image that reflected their emotions in that specific moment.
(h) At the end of this phase, the child started the dental session with one of two clinicians.

#### 2.4.2. Intervention

(a) The clinician decided, based on the clinical case and planned treatment, whether to use nitrous oxide/oxygen inhalation conscious sedation or to proceed using behavioral management techniques alone.
(b) Throughout the operating session, heart rate monitoring was performed using a pulse oximeter. The nursing staff recorded the data and subsequently handed them over to the clinician responsible for administering the test.
(c) Parents were allowed to remain in the room as passive observers and were instructed not to influence the child’s responses.

#### 2.4.3. Post-Operative Anxiety Assessment

(a) After leaving the operating room, the post-operative anxiety assessment was carried out.
(b) A set of eight color cards and the VFAS are administered following the same procedure as in the initial phase.

After the administration of both tests, the child was asked to draw a picture of the experience, thanking him for cooperative participation. The clinician consequently collected the data: the values obtained from the Lüscher test, from the VFAS, the heart rate values, the patient’s identification data (name, surname, sex, and age), the type of treatment undergone (invasive therapies: surgical therapy of the carious lesion and dental extraction with the administration of local anesthesia; non-invasive therapies: medical therapy of the carious lesion), the name of the clinician who performed the dental session and the use or not of nitrous oxide as the inhalation sedation technique.

N_2_O/O_2_ inhalation sedation was selected based on clinical judgment and patient cooperation and was not randomly allocated. In pediatric patients, inhalation sedation was administered via a nasal mask (sizes S or M). The gas mixture ranged between 35% and 70% nitrous oxide in oxygen. Sedation was initiated at 50% nitrous oxide and subsequently titrated in 5–10% increments or decrements according to the child’s clinical response, with a typical maximum of 70% during more critical clinical phases (e.g., local anesthesia administration). Continuous clinical monitoring was performed by the clinician and supported by pulse oximetry, with vital parameters observed throughout the procedure. At the end of the procedure, patients received 100% oxygen for 4 min. No adverse events were observed during the study procedures.

#### 2.4.4. Interpretation of Tests

The Lüscher Color Test was administered according to the standardized procedure. Scoring and interpretation were performed by a single trained examiner. The test was conducted in a quiet clinical room under standardized artificial lighting conditions. The eight color cards were presented simultaneously and arranged randomly on a flat surface in front of the child. Children were instructed to select the colors as quickly and instinctively as possible to capture their immediate emotional response. No strict time limit was imposed; however, participants were encouraged to respond promptly. Parents were allowed to remain in the room as passive observers and were instructed not to influence the child’s choices. Each child was asked to select and order the eight colored cards in descending order of preference, from the most to the least preferred. The test was administered twice (before and after dental treatment) with a short interval between administrations. Each color card bears a predefined numerical code on its reverse side. Based on the ordered sequence, color pairs were generated and their symbolic functions were identified according to the original interpretative framework: strong preference (+), moderate preference (x), indifference (=), and rejection (−). For quantitative analysis, a continuous anxiety-related index was derived from the ordered color sequence. Specifically, the positional ranks of the four basic colors (blue, green, red, and yellow) were used to compute a composite numeric score according to the Lüscher interpretative framework. Less favorable placement of these basic colors resulted in higher scores, which within the framework of the test were interpreted as reflecting greater anxiety-related features. The traditional exclamation-mark (!/!!/!!!) criteria were used solely to support clinical interpretation and were not directly analyzed statistically. The continuous composite score was used for all statistical analyses. To ensure procedural consistency, all tests were interpreted by the same trained examiner using a standardized scoring procedure. Appendix A.

### 2.5. Statistical Analysis

The data were entered into a spreadsheet (Excel; Microsoft, Inc., Redmond, WA, USA) throughout the study period. The primary outcome was the pre–post change in the Lüscher anxiety index. Effect sizes were reported as partial eta squared (ηp^2^) for repeated-measures ANOVA and Cohen’s dz for paired pre–post comparisons. As a pre-specified secondary analysis, agreement between subjective measures (Lüscher and VFAS change scores) and the physiological parameter (percentage HR reduction) was assessed using Spearman’s correlation. Subsequent models used the subjective measure showing the strongest agreement with HR. One-way ANOVA was performed to evaluate associations between independent variables (age, sex, clinician, nitrous oxide use, and type of treatment) and the selected dependent variable (Lüscher change score). Logistic regression analysis was then conducted to explore the relative contribution of independent variables. Variables were dichotomized before inclusion in the regression model, and backward selection was applied with a removal significance level of 0.1. A *p*-value < 0.05 was considered statistically significant. Power calculations were performed using SPSS (version 30). For logistic regression, the outcome was dichotomized as anxiety reduction (yes/no) to provide a clinically interpretable endpoint. Given the sample size and to reduce sparse data in some categories, selected predictors were dichotomized using clinically meaningful cutoffs and/or distribution-based thresholds. We acknowledge that dichotomization may reduce statistical power and information.

## 3. Results

**Figure 1:**
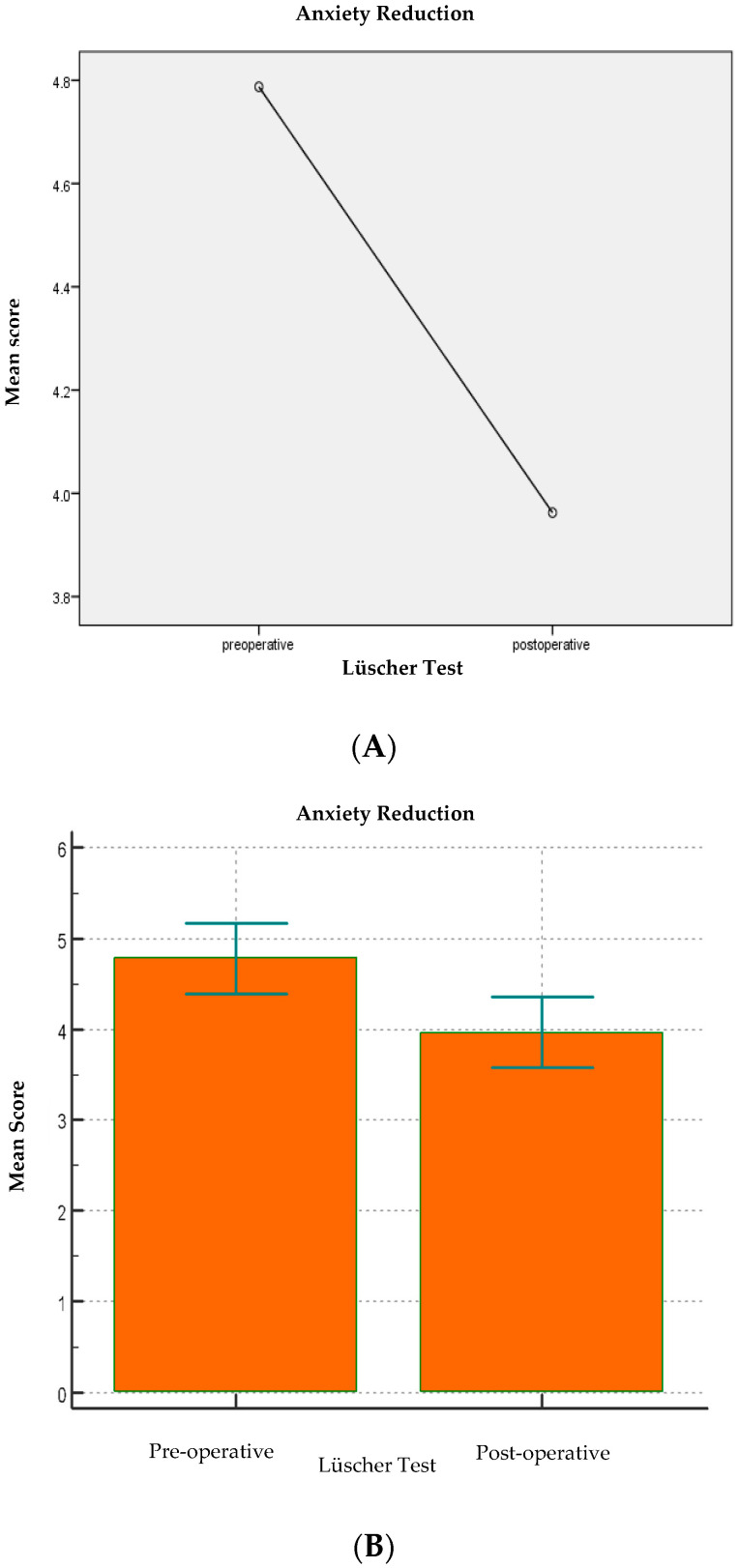
(**A**,**B**) Comparison of pre-operative vs. post-operative Lüscher score. Descriptive statistics and *p*-values. Abbreviations: SD, standard deviation; CI, confidence interval.

**Figure 2:**
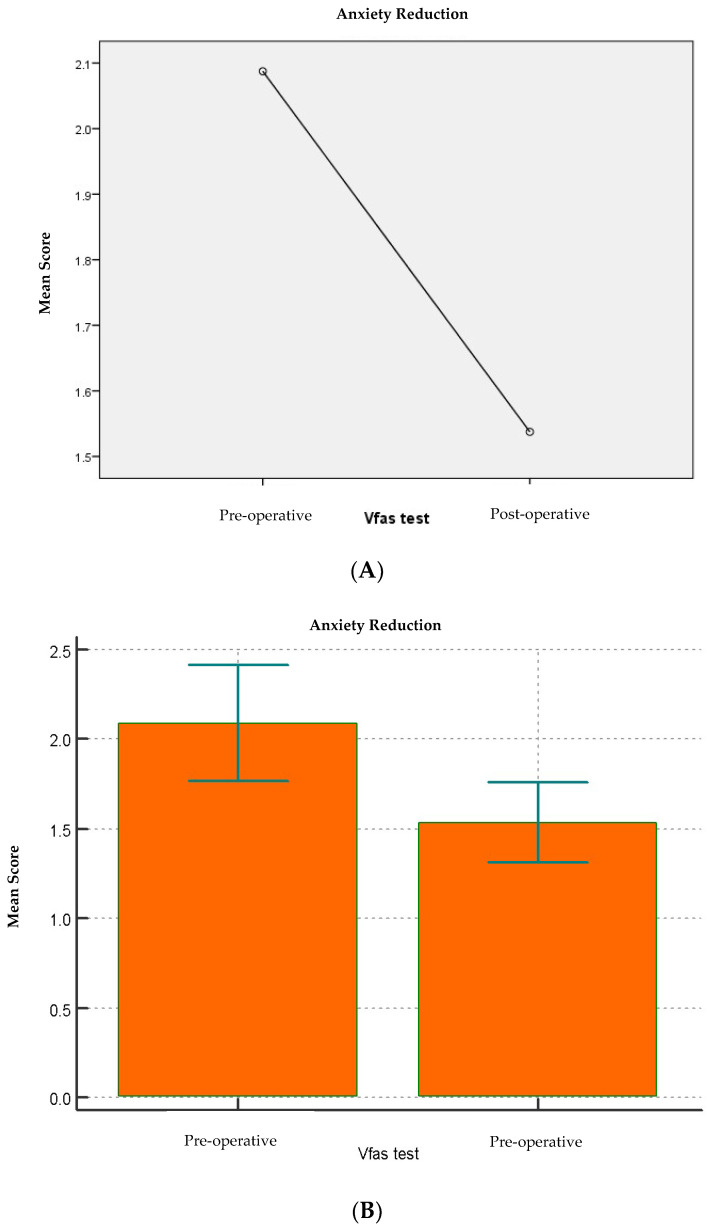
(**A**,**B**) Comparison of pre-operative vs. post-operative VFAS score. Descriptive statistics and *p*-values. Abbreviations: VFAS, Visual Facial Anxiety Scale; SD, standard deviation; CI, confidence interval.

Of the 80 patients in the sample, 40 patients were treated with nitrous oxide at the clinician’s discretion, and 40 were managed using behavioral management techniques alone. A total of 46 were males and 34 females, aged between 4 and 12 years (mean 6.5 ± 1.6 SD). After collecting the general data through the medical record, the level of anxiety before and after dental treatment was measured, using the Lüscher color test, the VFAS, and the pulse oximeter for heart rate values as diagnostic tools. Data were analyzed according to sex, age, type of treatment, use of nitrous oxide, the clinician who performed the dental session, and the level of anxiety reported before and after dental treatment. The descriptive statistics of the sample are shown in Table 1. According to age criteria, the patients were grouped under six years old (n = 42) and over six years old (n = 38). A total of 40 invasive treatments and 40 non-invasive treatments were carried out. Clinician 1 performed 35 operations, while clinician 2 performed 45. The pre-operative heart rate was 101.7 bpm (±12.3) while the pre-operative score of the Lüscher test was 4.8 (±1.7) and the pre-operative VFAS score 2.1 (±1.4). There was no statistically significant difference between the nitrous oxide and control groups concerning pre-operational variables. Baseline anxiety values were elevated according to both instruments (Lüscher and VFAS) [Table 2 and Table 3; Figure 1 and Figure 2].

Overall, anxiety decreased after treatment. Lüscher anxiety scores decreased from pre- to post-operative (pre: 4.79 ± 1.76; post: 3.96 ± 1.75), F(1,79) =20.53, *p* < 0.001, ηp^2^ = 0.21; the corresponding paired effect size was Cohen’s dz = 0.51. The mean change was ∆Lüscher (pre − post) = 0.82. VFAS scores also showed a significant reduction (mean change = 0.60, *p* < 0.001, ηp^2^ = 0.10).

A multivariable logistic regression analysis was conducted to evaluate predictors of anxiety reduction (yes/no). The model included age group, sex, clinician, type of treatment (invasive vs. non-invasive), and nitrous oxide use. Model fit indices indicated modest performance. The McFadden pseudo-R^2^ was 0.059, suggesting limited explanatory power consistent with the exploratory design of the study. The model demonstrated fair discriminative ability, with an area under the receiver operating characteristic curve (AUC) of 0.66 and an overall classification accuracy of 65%. Among the variables included in the model, nitrous oxide use showed a trend toward association with anxiety reduction. Children who did not receive nitrous oxide had higher odds of no anxiety reduction (OR = 2.33); however, this association did not reach statistical significance (*p* = 0.07).

**Figure 3:**
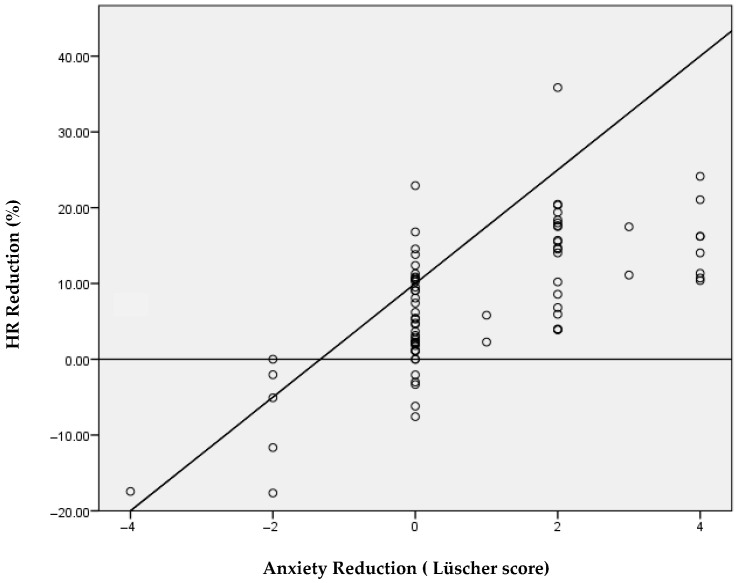
Spearman’s coefficient for assessing the correlation between HR score and Lüscher test. Abbreviations: HR, heart rate; r, Spearman’s correlation coefficient.

**Figure 4:**
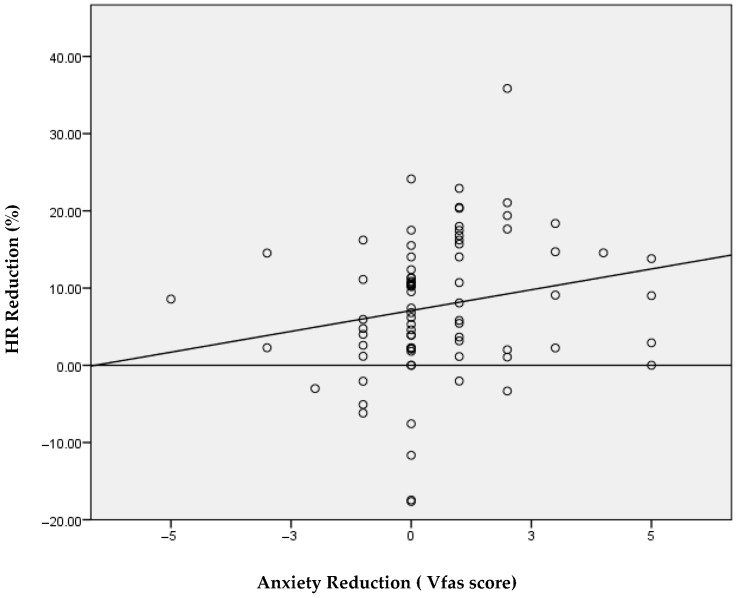
Spearman’s coefficient for assessing the correlation between HR score and VFAS. Abbreviations: VFAS, Visual Facial Anxiety Scale; HR, heart rate; r, Spearman’s correlation coefficient.

To evaluate agreement between subjective anxiety measures and the physiological parameter, we calculated Spearman’s correlations between the percentage reduction in heart rate (HR) and the pre–post change scores of both instruments. A strong, statistically significant correlation was observed between HR reduction and the change in Lüscher scores (r = 0.68; *p* < 0.01; Table 4 and Figure 3). In contrast, the correlation between HR reduction and the change in VFAS scores was weak, although statistically significant (r = 0.28; *p* < 0.05; Table 4 and Figure 4). Overall, the Lüscher Color Test showed substantially higher agreement with the physiological measure compared with the VFAS.

**Figure 5:**
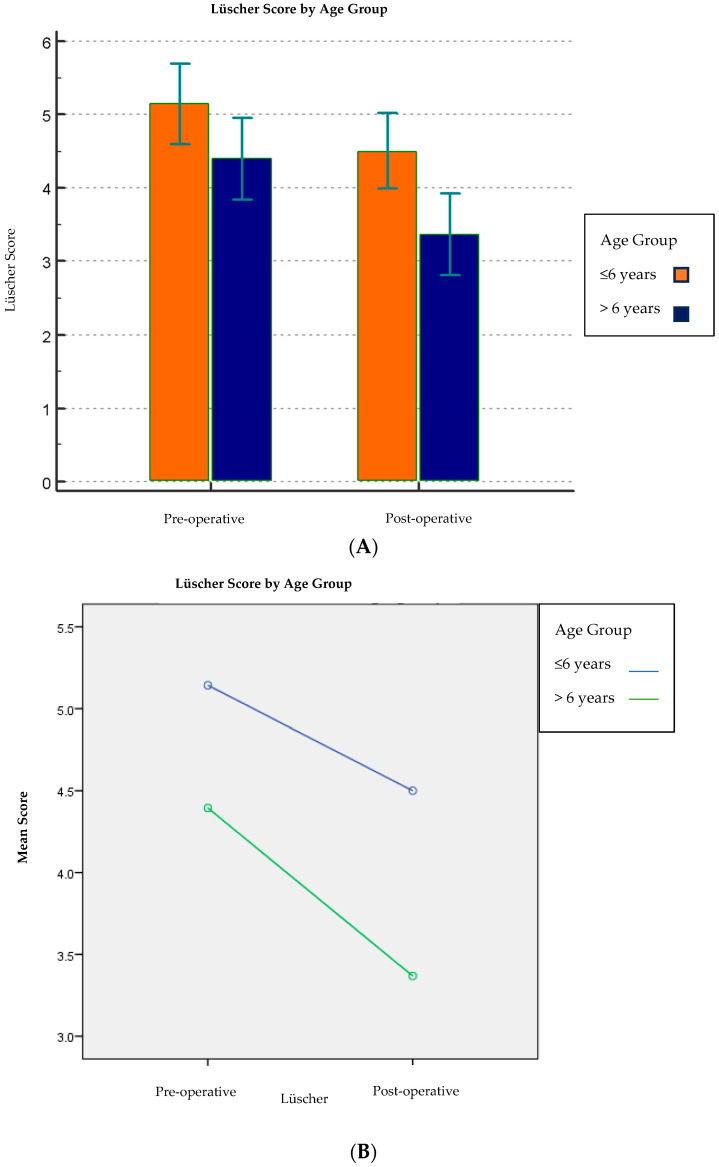
(**A**,**B**) Comparison of the Lüscher score between the two age groups. Abbreviations: SD, standard deviation; CI, confidence interval.

**Figure 6:**
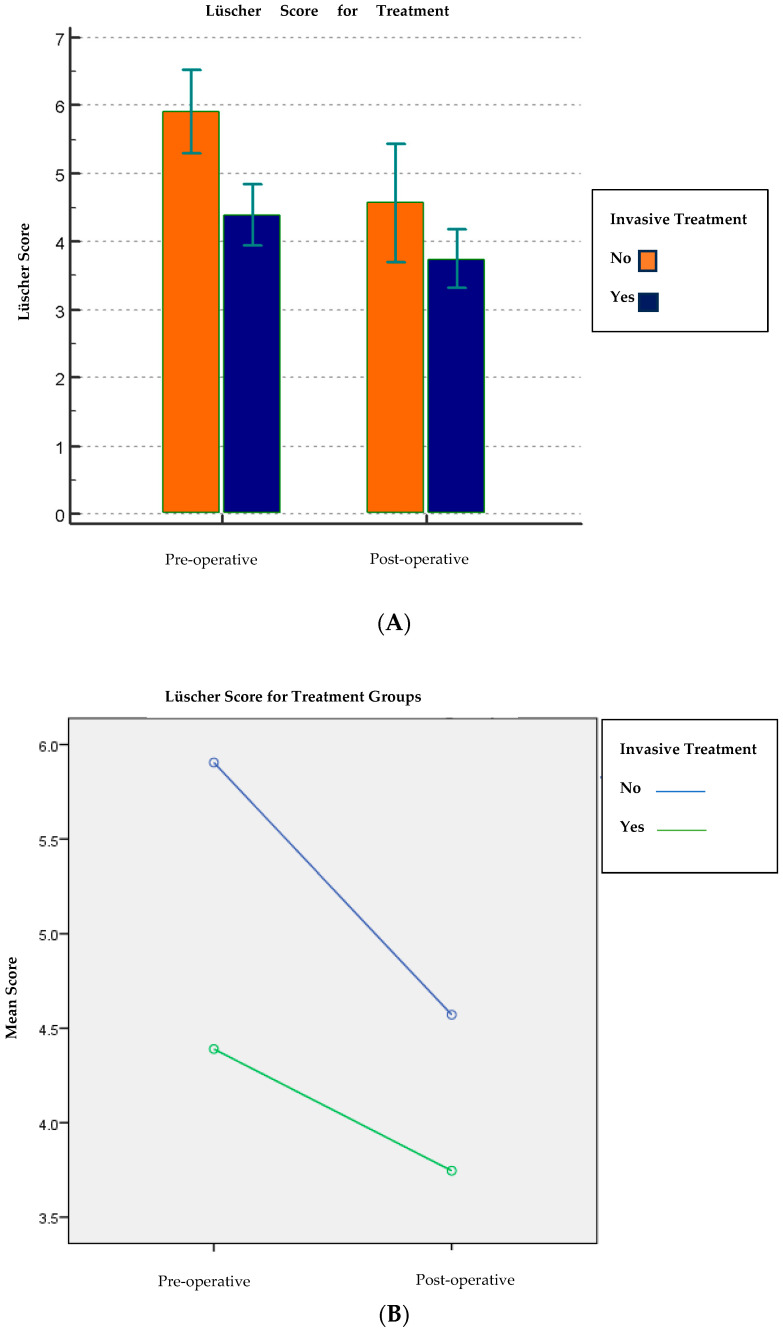
(**A**,**B**) Comparison of the Lüscher score between the two treatment groups. Abbreviations: SD, standard deviation; CI, confidence interval.

**Figure 7:**
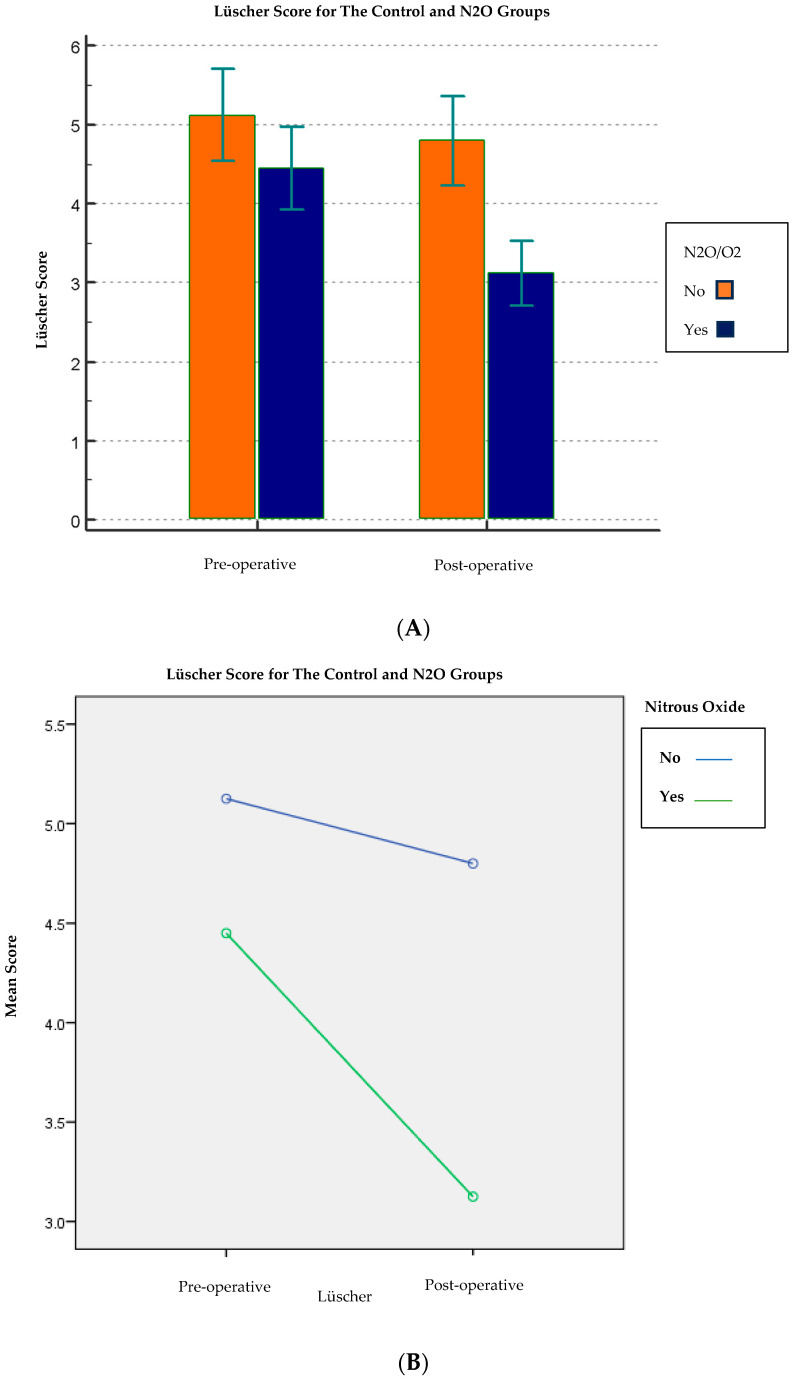
(**A**,**B**) Lüscher scores comparison between N_2_O/O_2_ and control group. Abbreviations: N_2_O/O_2_, nitrous oxide/oxygen; SD, standard deviation; CI, confidence interval.

The pre-operative Lüscher score (4.8 ± 1.8) was found to be statistically highly significant (*p* < 0.001) compared to the post-operative Lüscher score (4.0 ± 1.7) [Table 2; Figure 1]. Furthermore, of all the independent variables under investigation, the age variable (*p* = 0.006), the treatment variable (*p* = 0.002), and then the variable use or non-use of nitrous oxide (*p* < 0.001) were statistically significant about the difference between pre- and post-operative scores. On the other hand, the sex variable and the clinician variable were not statistically significant concerning the pre- and post-operative score (*p* = 0.36 and *p* = 0.50, respectively) [Table 5; Figure 5, Figure 6 and Figure 7].

Children who did not receive nitrous oxide treatment showed higher odds of no anxiety reduction (OR = 2.33); however, this association did not reach statistical significance (*p* = 0.07) [Table 6]. A very significant result is that out of 80 cases, there was a worsening of anxiety from pre- to post-operative with worsening anxiety in six cases. In all six cases in which a worsening of anxiety was found, the children were treated without nitrous oxide and invasive treatments.

## 4. Discussion

### 4.1. Correlations

The present prospective clinical study explored the applicability of a non-verbal tool (the Lüscher Color Test) for assessing anxiety-related changes in behaviorally challenging pediatric patients treated in a conscious sedation setting. Lüscher score values should be interpreted within the test’s own framework and are not diagnostic of anxiety; instead, they reflect anxiety-related features at a specific time point.

In our sample, both subjective tools showed a reduction in pre–post anxiety scores (Lüscher: 4.8 ± 1.8 to 4.0 ± 1.7, *p* < 0.001; VFAS: 2.1 ± 1.5 to 1.5 ± 1.0). Importantly, the magnitude of association with physiological change differed between the two subjective measures: the pre–post change in Lüscher scores was strongly associated with HR reduction (Spearman r = 0.68, *p* < 0.01), whereas VFAS change showed only a weak association (r = 0.28, *p* < 0.05). These observations should not be interpreted as evidence against the validity of VFAS, which is a validated pediatric anxiety scale. Rather, in our specific clinical context (young and behaviorally challenging children), VFAS responses may have been influenced by developmental factors, response style, or situational preferences. Therefore, VFAS should be considered an established comparator, while the Lüscher Color Test remains exploratory in this study.

This discrepancy may reflect limitations of brief facial self-report measures in young or distressed children. During administration, some children appeared to select faces based on preference (e.g., choosing the “happiest” face) or social desirability, and younger children occasionally looked to parents for approval. These qualitative observations are consistent with previous reports that facial image scales may be influenced by response style and developmental factors. However, these findings should not be interpreted as evidence that VFAS is invalid, but rather that its performance may be context-dependent in this specific clinical population [9,10,11,12,13,14].

Heart rate interpretation: HR is a non-specific physiological marker of autonomic arousal and can be influenced by multiple factors beyond anxiety, including procedural stimulation, pain/discomfort, respiratory changes, baseline autonomic tone, and pharmacophysiological effects of N_2_O/O_2_ inhalation sedation. Therefore, HR reduction should not be interpreted as a pure proxy for anxiety reduction in this setting, but rather as a supportive physiological correlation that may partially reflect procedural and sedation-related influences. Accordingly, the observed association between Lüscher score changes and HR reduction should be interpreted as exploratory and not as evidence of diagnostic or construct validity. Formal validation would require dedicated psychometric studies assessing reliability, construct validity, criterion validity, and responsiveness.

### 4.2. Age-Related Differences

Age is widely considered a relevant factor in pediatric dental anxiety, with younger children often displaying higher anxiety levels. In our study, children ≤ 6 years showed higher pre-operative anxiety scores than older children (>6 years) (5.1 ± 1.7 vs. 4.4 ± 1.7). Both groups showed a reduction after treatment, with a greater reduction observed in older children (4.4 ± 1.7 to 3.4 ± 1.7) compared to younger children (5.1 ± 1.7 to 4.5 ± 1.6; *p* = 0.006). These findings are in line with developmental explanations, such as fear of unfamiliar situations and limited coping strategies in younger children [15].

### 4.3. Gender and Clinician Variables

In our sample, gender was not significantly associated with anxiety reduction (*p* = 0.36), consistent with literature suggesting inconsistent or negligible gender effects on dental anxiety outcomes. Although males showed slightly higher pre- and post-operative anxiety values, these differences were not statistically significant and may reflect variability rather than systematic effects [16].

Similarly, clinician-related differences were not statistically significant (*p* = 0.50). This may be explained by the similar clinical training and operative approach of both clinicians. Given the limited sample and the observational nature of the study, small clinician effects cannot be excluded and could be better assessed in larger cohorts with standardized behavioral and communication measures.

### 4.4. Treatment Type

In the bivariate comparisons, children receiving non-invasive treatment showed higher pre- and post-operative anxiety values than those receiving invasive treatment (pre 5.9 ± 1.3 vs. 4.4 ± 1.7; post 4.6 ± 1.9 vs. 3.7 ± 1.6; *p* = 0.002). This finding may reflect case-mix differences and pre-existing anxiety profiles rather than procedure invasiveness alone.

However, in the logistic regression model, treatment type was not significantly associated with anxiety reduction. Among children undergoing invasive treatment (n = 59), anxiety reduction was observed in 23 cases (OR = 1.22), while among those undergoing non-invasive treatment (n = 21), anxiety reduction occurred in 9 cases (reference; OR = 1.00; *p* = 0.71). The imbalance in subgroup sizes may have reduced statistical power to detect differences for this predictor [17].

### 4.5. Nitrous Oxide (N_2_O/O_2_)

Children in the control group showed higher pre-operative anxiety than those receiving N_2_O/O_2_ (5.1 ± 1.8 vs. 4.4 ± 1.6), and post-operative anxiety followed a similar pattern (4.8 ± 1.8 vs. 3.1 ± 1.3), with a larger pre–post reduction observed in the N_2_O/O_2_ group (*p* < 0.001).

In the multivariable logistic regression, nitrous oxide showed a non-significant trend toward association with anxiety reduction (*p* = 0.07), which does not meet the conventional threshold for statistical significance (α = 0.05).

Because sedation allocation was based on clinical judgment rather than randomization, this finding is susceptible to selection bias and confounding by indication. Accordingly, no causal inference can be drawn regarding the effect of nitrous oxide on anxiety reduction, and the result should be interpreted as hypothesis-generating.

Moreover, nitrous oxide may influence HR through physiological mechanisms, further limiting the interpretation of HR reduction as anxiety-specific [18,19,20].

### 4.6. Multivariable Model Performance

The overall logistic regression model showed modest performance (McFadden pseudo-R^2^ = 0.059; AUC = 0.66), indicating limited explanatory power and fair discriminative ability. These findings are consistent with the exploratory observational design and the relatively small sample size. Therefore, the regression results should be interpreted cautiously and considered hypothesis-generating rather than confirmatory.

### 4.7. Reliability and Limitations Specific to Lüscher Interpretation

The Lüscher Color Test relies on an interpretative psychological framework, and its psychometric robustness in pediatric dental settings has not been firmly established. Accordingly, the present findings should be considered exploratory and hypothesis-generating.

The test is grounded on the theoretical assumption that color preferences may reflect underlying emotional and psychophysiological states. Within its original framework, the relative positioning of specific colors is interpreted as providing indirect indicators of an individual’s current affective condition. However, the construct validity and psychometric properties of this approach have been questioned in psychological literature. While the test has been widely used in clinical and psychological contexts, its application in pediatric dentistry has been only marginally investigated. In this field, rapid non-verbal instruments may be particularly useful for assessing anxiety in young or poorly cooperative children; therefore, this study focuses on exploring potential clinical applicability rather than providing formal validation [21,22,23,24,25,26].

A further limitation concerns the interpretative component of scoring. In the present study, scoring and interpretation were performed by a single examiner, and inter-rater reliability was not assessed, which may limit reproducibility. Future studies should include examiner calibration and formal reliability testing, as well as validation analyses against established pediatric dental anxiety measures and longitudinal outcomes [27,28,29].

## 5. Strengths and Limitations

This study has several strengths. First, it addresses a clinically relevant and still underexplored topic in pediatric dentistry: the assessment of dental anxiety in uncooperative children using a non-verbal psychological tool. The prospective design and the combined use of subjective and objective anxiety-related measures represent important methodological advantages. Heart rate monitoring provided a physiological parameter that allowed comparison with psychological assessment tools in a real clinical setting.

A further strength is the standardized administration of both the Lüscher Color Test and the Visual Facial Anxiety Scale (VFAS) before and after treatment, which enabled evaluation of anxiety-related changes associated with the dental experience. In addition, the study population consisted of behaviorally challenging pediatric patients treated in a conscious sedation setting, increasing the clinical relevance of the findings and reflecting routine practice conditions.

Another strength is the inclusion of multiple demographic and clinical variables (including age, treatment type, clinician, and N_2_O/O_2_ inhalation sedation), which allowed a broader exploratory analysis of factors potentially associated with anxiety reduction. Finally, the high acceptability of the Lüscher Color Test among children supports its feasibility as a complementary non-verbal approach in this population.

At the same time, several limitations should be acknowledged. First, the sample size was relatively small and derived from a single center, which may limit the generalizability of the findings. Although baseline characteristics were broadly comparable, larger multicenter studies are needed to confirm these results.

Second, N_2_O/O_2_ inhalation sedation was not randomly allocated but selected according to clinical judgment. This introduces the possibility of selection bias and confounding by indication, and therefore no causal inference can be drawn regarding the effect of sedation on anxiety reduction.

Third, neither test administration nor Lüscher interpretation was blinded, which may have introduced observer and interpretation bias. In addition, the interpretative nature of the Lüscher Color Test represents a relevant limitation: although the test showed an association with physiological and behavioral indicators of anxiety, its construct validity and reproducibility in pediatric dental settings remain uncertain. Lüscher interpretation was performed by a single trained examiner, and inter-rater reliability was not assessed, which may further affect reproducibility.

Fourth, the VFAS relied exclusively on child self-report. In younger children, responses may have been influenced by developmental factors, parental presence, or preference for facial expressions, potentially affecting response consistency in this specific clinical context. However, this should not be interpreted as a limitation of the VFAS as a validated pediatric anxiety scale, but rather as a context-related issue in this sample.

Fifth, subgroup imbalance (invasive procedures, *n* = 59, vs. non-invasive procedures, *n* = 21) may have reduced statistical power, particularly in regression analyses, contributing to wider confidence intervals and non-significant associations for some variables. For the same reason, the multivariable regression results should be interpreted as exploratory and hypothesis-generating.

Finally, anxiety was assessed during a single treatment session only, without longitudinal follow-up across repeated visits. Therefore, the findings should be considered preliminary and do not allow conclusions about the stability of anxiety responses over time or the cumulative effect of repeated dental care experiences. Future studies should include repeated assessments and formal psychometric validation to better define the clinical role of non-verbal anxiety assessment tools.

## 6. Conclusions

The aim of this study was to assess the clinical applicability of administering the Lüscher Color Test in behaviorally challenging pediatric patients treated under conscious sedation and to explore its association with established anxiety proxies (VFAS and heart rate changes). In line with the null hypothesis that the Lüscher Color Test would not show stronger agreement with physiological arousal than a facial self-report scale, our results showed that the Lüscher pre–post change score was more strongly associated with heart rate reduction than the VFAS in this sample. However, because heart rate is a non-specific physiological marker and sedation was not randomized, these findings should be interpreted as observational associations rather than evidence of validity or causal effects.

Regarding treatment-related predictors, the multivariable logistic regression did not demonstrate statistically significant associations for treatment type, and nitrous oxide showed only a non-significant trend (*p* = 0.07). Overall, the Lüscher Color Test appeared feasible and acceptable to children in this setting, but its interpretation remains limited by the lack of reliability testing and the ongoing controversy regarding its psychometric properties. Further studies designed specifically for validation (including reliability assessment, comparison with validated pediatric dental anxiety instruments, and larger samples) are needed before recommending routine clinical use.

## Figures and Tables

**Table 1:** Descriptive statistics of pre-operative variables for the study sample. Data presented as mean (SD) or ratio. There were no statistically significant differences between the nitrous oxide (N_2_O/O_2_) and control groups.

	Control (n = 40)	N_2_O/O_2_ (n = 40)	Total (80)	*p*-Values
Age (years)	6.4 (1.6)	6.6 (1.6)	6.5 (1.6)	0.65
Age (0–6/>6 years)	22/18	20/20	42/38	0.65
Sex (Males/Females)	22/18	24/16	46/34	0.65
Invasive treatment (yes/no)	28/12	31/9	40/40	0.45
Clinician (1/2)	23/17	22/18	35/45	0.82
pre-operative HR	102.7 (13.8)	100.7 (10.7)	101.7 (12.3)	0.34
pre-operative Lüscher score	5.1 (1.8)	4.4 (1.6)	4.8 (1.7)	0.07
pre-operative VFAS score	1.8 (1.3)	2.4 (1.6)	2.1 (1.4)	0.08

Abbreviations: N_2_O/O_2_, nitrous oxide/oxygen; SD, standard deviation; HR, heart rate; VFAS, Visual Facial Anxiety Scale.

**Table 2:** Comparison of pre-operational vs. post-operational Lüscher score. Descriptive statistics and *p*-value (analysis of variance of repeated measures—ANOVA).

Lüscher Score	Mean (SD)	CI 95%	*p*-Value
pre-operative	4.8 (1.8)	4.4–5.2	<0.001
post-operative	4.0 (1.7)	3.6–4.3	

Abbreviations: SD, standard deviation; CI, confidence interval; ANOVA, analysis of variance.

**Table 3:** Comparison of pre-operational vs. post-operational Visual Facial Anxiety Scale (VFAS) score. Descriptive statistics and *p*-value (analysis of variance of repeated measures—ANOVA).

VFAS Score	Mean (SD)	CI 95%	*p*-Value
pre-operative	2.1 (1.5)	1.8–2.4	<0.001
post-operative	1.5 (1.0)	1.3–1.8	

Abbreviations: VFAS, Visual Facial Anxiety Scale; SD, standard deviation; CI, confidence interval; ANOVA, analysis of variance.

**Table 4:** Spearman’s coefficient correlation between HR value and pre- and post-operative anxiety reduction values with Lüscher/VFAS.

Spearman R		
HR Reduction	VFAS Reduction	Lüscher Scale Reduction
%	0.28 *	0.68 **

** *p* < 0.01; * *p* < 0.05. Abbreviations: HR, heart rate; r, Spearman’s correlation coefficient. Note: Spearman’s rho correlations are between percentage heart rate reduction and the pre–post change scores (Δ) of Lüscher and VFAS.

**Table 5:** Comparison of Lüscher score between groups. Descriptive statistics and *p*-values (analysis of variance of repeated measures—ANOVA).

Lüscher Score					
	Pre-Operative		Post-Operative		
	Mean (SD)	CI 95%	Mean (SD)	CI 95%	*p*-Value
Age					0.006
≤6 years	5.1 (1.7)	4.6–5.7	4.5 (1.6)	4.0–5.0	
>6 years	4.4 (1.7)	3.8–4.9	3.4 (1.7)	2.8–3.9	
Sex					0.36
M	5.0 (1.6)	4.5–5.5	4.0 (1.8)	3.3–4.5	
F	4.4 (1.8)	3.8–5.1	3.9 (1.7)	3.4–4.5	
Invasive Treatment					0.002
no	5.9 (1.3)	5.3–6.5	4.6 (1.9)	3.7–5.4	
yes	4.4 (1.7)	3.9–4.8	3.7 (1.6)	3.3–4.1	
Clinician					0.50
1	4.9 (1.8)	4.3–5.4	4.1 (1.7)	3.6–4.6	
2	4.7 (1.7)	4.1–5.3	3.8 (1.9)	3.2–4.4	
N_2_O/O_2_					<0.001
no	5.1 (1.8)	4.5–5.7	4.8 (1.8)	4.2–5.4	
yes	4.4 (1.6)	3.9–5.0	3.1 (1.3)	2.7–3.5	

**Table 6:** Risk factors for no reduction in anxiety. Logistic regression analysis: selection (P-to-remove = 0.1). Odds ratio (OR), 95% confidence interval (CI), and *p*-value.

Lüscher Score							
Independent Variables	Category	Total Number	Si Anxiety Reduction = 0 Number	No Anxiety Reduction = 1 Number	Odds Ratio	CI 95%	*p*-Value
Age	≤6 years	42	16	26	1.25	0.49–3.19	0.64
	>6 years	38	16	22	1.00		
Sex	M	46	22	24	1.00	0.84–5.66	0.11
	F	34	10	24	2.17		
Invasive Treatment	No	21	9	12	1.00	0.42–3.35	0.71
	Si	59	23	36	1.22		
Clinician	1	45	18	27	1.00	0.40–2.63	0.96
	2	35	14	21	1.03		
Nitrous oxide	No	40	12	28	2.33	0.93–5.84	0.07
	Si	40	20	20	1.00		

Abbreviations: OR, odds ratio; CI, confidence interval.

## Data Availability

The data presented in this study are available on request from the corresponding author. The data are not publicly available due to privacy and ethical reasons.

## Appendix A

This appendix is provided in order to enhance transparency and reproducibility. It includes additional methodological information and supporting details that complement Section 2.

## References

[1] Sun I.G., Chu C.H., Lo E.C.M., Duangthip D. (2024). Global prevalence of early childhood dental fear and anxiety: A systematic review and meta-analysis. J. Dent..

[2] Cianetti S., Lombardo G., Lupatelli E., Pagano S., Abraha I., Montedori A., Caruso S., Gatto R., De Giorgio S., Salvato R. (2017). Dental Fear/Anxiety Among Children and Adolescents. A Systematic Review. Eur. J. Paediatr. Dent..

[3] Firetto M.C., Abbinante A., Barbato E., Bellomi M., Biondetti P., Borghesi A., Bossu’ M., Cascone P., Corbella D., Di Candido V. (2019). National Guidelines for Dental Diagnostic Imaging in the Developmental Age. Radiol. Med..

[4] Hussain A., Heather B., Paul A. (2018). Dentists’ use of validated child dental anxiety measures in clinical practice: A mixed-methods study. Int. J. Paediatr. Dent..

[5] Barry M., Alnami M., Alshobaili Y.T., Felemban O.M., Sabbagh H.J. (2025). Assessment of Dental Fear and Anxiety Tools for Children: A Review. Healthcare.

[6] Picco R.D., Dzindolet M.T. (1994). Examining the Lüscher Color Test. Percept. Mot. Ski..

[7] Lee S., Westland S., Rodski S. (2024). Exploring colour choice rank order and its relationship with heart rate variability. J. Int. Colour Assoc..

[8] Sanglard L.F., Oliveira L.B., Massignan C., Polmann H., De Luca Canto G. (2022). Evaluating Pain, Fear, Anxiety or Stress/Distress Using Children’s Drawings in Paediatric Dentistry: A Scoping Review. Eur. Arch. Paediatr. Dent..

[9] Klar H. (1965). Die verwendung des lüscher-tests in der ärztlichen praxis. schlussworte [application of the lüscher test in medical practice. Concluding comments]. Med. Welt..

[10] Farronato G., Giannini L., Galbiati G., Maspero C. (2011). Modified Hyrax expander for the correction of upper midline deviation: A case report. Minerva Stomatol..

[11] El-Housseiny A., Farsi N., Alamoudi N., Bagher S., El Derwi D. (2014). Assessment for the Children’s Fear Survey Schedule–Dental Subscale. J. Clin. Pediatr. Dent..

[12] Nirmala S.V.S.G., Veluru S., Nuvvula S., Chilamakuri S. (2015). Preferences of Dentist’s Attire by Anxious and Nonanxious Indian Children. J. Dent. Child..

[13] BaniHani A., Santamaría R.M., Hu S., Maden M., Albadri S. (2022). Minimal Intervention Dentistry for Managing Carious Lesions into Dentine in Primary Teeth: An Umbrella Review. Eur. Arch. Paediatr. Dent..

[14] Santana M.D., de Souza A.C., de Abreu L., Valenti V.E. (2013). Association Between Oral Variables and Heart Rate Variability. Int. Arch. Med..

[15] Le S.H., Tonami K., Umemori S., Nguyen L.T.-B., Ngo L.T.-Q., Mataki S. (2018). The Potential of Heart Rate Variability for Exploring Dental Anxiety in Mandibular Third Molar Surgery. Int. J. Oral Maxillofac. Surg..

[16] Ludovichetti F.S., de Freitas J.G., Manera C., Lucchi P., Giordano F.I., Stellini E., Mazzoleni S. (2025). Assessing Pediatric Dental Stress Through Wearable Technology: Influence of Procedure Type, Treatment Phase, and Age. Eur. J. Dent..

[17] Dhaliwal J.K., Alzyood M., Al Rawashdeh R. (2025). A systematic review and narrative synthesis of evidence from randomised controlled trials: The impact of behaviour guidance techniques on dental anxiety in paediatric patients. BMC Oral Health.

[18] Arrow P., Klobas E. (2015). Minimum Intervention Dentistry Approach to Managing Early Childhood Caries: A Randomized Control Trial. Community Dent. Oral Epidemiol..

[19] Simon A.K., Bhumika T.V., Nair N.S. (2015). Does Atraumatic Restorative Treatment Reduce Dental Anxiety in Children? A Systematic Review and Meta-Analysis. Eur. J. Dent..

[20] AAPD (2025). Use of nitrous oxide for pediatric dental patients. The Reference Manual of Pediatric Dentistry 2023, Latest Revision 2023.

[21] Rossit M., Gil-Manich V., Ribera-Uribe J.M. (2021). Success rate of nitrous oxide-oxygen procedural sedation in dental patients: Systematic review and meta-analysis. J. Dent. Anesth. Pain Med..

[22] Kebriaee F., Sarraf Shirazi A., Fani K., Moharreri F., Soltanifar A., Khaksar Y., Mazhari F. (2015). Comparison of the Effects of Cognitive Behavioural Therapy and Inhalation Sedation on Child Dental Anxiety. Eur. Arch. Paediatr. Dent..

[23] Alasmari A.A., Aldossari G.S., Aldossary M.S. (2018). Dental Anxiety in Children: A Review of the Contributing Factors. J. Clin. Diagn. Res..

[24] Esposito L., Poletti L., Maspero C., Porro A., Pietrogrande M.C., Pavesi P., Dellepiane R.M., Farronato G. (2012). Hyper-IgE syndrome: Dental implications. Oral Surg. Oral Med. Oral Pathol. Oral Radiol..

[25] Wu L., Gao X. (2018). Children’s Dental Fear and Anxiety: Exploring Family-Related Factors. BMC Oral Health.

[26] Levesque J., Ghotra S., Mittermuller B.A., DeMaré D., Lee V.H.K., de Jesus V.C., Olatosi O.O., Alai-Towfigh H., Schroth R.J. (2024). Canadian Dentists’ Awareness and Views on Early Childhood Caries and Its Prevention and Management. Front. Oral Health.

[27] Farronato G., Giannini L., Galbiati G., Maspero C. (2014). A 5-year longitudinal study of survival rate and periodontal parameter changes at sites of dilacerated maxillary central incisors. Prog. Orthod..

[28] Carrillo-Díaz M., Migueláñez-Medrán B.C., Nieto-Moraleda C., Romero-Maroto M., González-Olmo M.J. (2021). How Can We Reduce Dental Fear in Children? The Importance of the First Dental Visit. Children.

[29] Maspero C., Giannini L., Riva R., Tavecchia M.G., Farronato G. (2009). Nasal cycle evaluation in 10 young patients: Rhynomanometric analysis. Mondo Ortod.

